# Burden of congenital and hereditary anomalies in the war-affected territory at Pakistan–Afghanistan border

**DOI:** 10.2478/abm-2022-2033

**Published:** 2023-08-01

**Authors:** Muhammad Naeem, Bashir Ahmad, Sajid Malik

**Affiliations:** Human Genetics Program, Department of Zoology, Faculty of Biological Sciences, Quaid-i-Azam University, Islamabad 45320, Pakistan

**Keywords:** birth defects, consanguinity, internally displaced persons, limb defects, neurological disorders

## Abstract

**Background:**

Pashtun populations of Pakistan are the victim of long-lasting military combats, rendering 1.9 million inhabitants internally displaced. Studies highlighting congenital and hereditary anomalies in these populations are deficient.

**Objectives:**

To elucidate the spectrum anomalies in the north-western war-affected territories of Pakistan.

**Methods:**

A cross-sectional study was carried out from 2017 to 2019 and individuals or families with anomalies were ascertained through convenience and cluster random sampling. Phenotypic and pedigree data and information on bio-demographic variables were collected. Descriptive statistics were applied.

**Results:**

A total of 361 independent individuals or families with anomalies were recruited. The anomalies were grouped into 8 major and 72 minor entities. Among major categories, neurological disorders had the highest representation (n = 100; proportion: 0.277; 95% CI: 0.231–0.323), followed by sensorineural defects (n = 70; prop.: 0.194), limb defects (n = 60; prop.: 0.166), visual impairments (n = 55; prop.: 0.152), and musculoskeletal defects (n = 37; prop.: 0.102). Among the neurological disorders, intellectual disability had the highest occurrence (58%), whereas talipes and limb amputations were the most prominent in limb defects (22% and 20%, respectively). The anomalies had sporadic and isolated presentations most often (76% each), while parental consanguinity was observed in 34% of index cases.

**Conclusions:**

The high incidence of neurological, sensorineural, and limb defects, the preponderance of sporadic cases, and low level of parental consanguinity may indicate a potentially high contribution of nongenetic factors in the etiology of anomalies. The majority of anomalies are the cause of severe disability.

Congenital anomalies (here referred to as anomalies) can be defined as structural or functional abnormalities that may be identified prenatally, at birth, or later in life and may tend to transmit in generations (i.e., be hereditary). The anomalies are the leading cause of infant and childhood mortality, disability, and chronic illnesses [1]. The anomalies are individually rare but collectively they make a significant impact on the populations. The prevalence of anomalies varies from country to country and from population to population [1, 2, 3].

Pakistan bears a high burden of anomalies [3, 5, 6, 7]. Hussain et al. [3] carried out a prospective hospital-based study at a tertiary care hospital in Kharian, Pakistan and witnessed that out of 3210 admissions, 226 (7%) neonates had a certain type of anomaly. Perveen and Tyyab [4] conducted a cross-sectional observational study at the Gynecology and Obstetrics department of a tertiary care hospital in Karachi, Pakistan, and observed that among the 5776 deliveries, 76 had babies with anomalies, rendering a prevalence rate of 11.4/1000 births. The authors further observed that consanguineous marriage was the most frequently associated risk factor for anomalies. Gustavson [5] studied the health and development of children in 4 different areas in Lahore and witnessed that the incidence of serious birth defects was 5%.

High-risk pregnancies are common in Pakistan due to the lack of education and awareness and antenatal care. Further, large family size and extended sibships, overlapping generations, and high consanguinity are the factors that increase the likelihood of occurrence of recessively inherited disorders [4, 6, 7]. There is, however, a lack of baseline epidemiological and phenotypic data on anomalies prevalent in various population strata. This aspect is further challenged by the absence of anomaly registries, lack of genetic counseling services, and inadequate resources. Epidemiological studies are particularly difficult to carry out in regions with geopolitical unrest and regional conflicts, and undergoing post-war recovery [8, 9, 10, 11].

The Pashtun populations of Pakistan inhabiting northwestern territories along the Afghanistan border are the victim of war-on-terror. Long-lasting military combats, cross-border infiltration, and poor law-and-order situation have rendered at least 1.9 million individuals internally displaced persons (IDPs) and gravely impacted the health and education systems [9, 11, 12, 13]. Even though the military operations are now over and the majority of the IDPs have returned to their “places,” it may take time to revive the socio-economic infrastructure and well-being of inhabitants. Studies highlighting morbidities of chronic nature and long-term illnesses including anomalies are lacking [11, 14]. To this end, through a cross-sectional study design, this study aimed to elucidate the spectrum and prevalence pattern of anomalies in the north-western territories of Pakistan.

## Methods

### Study population

**Figure 1.**
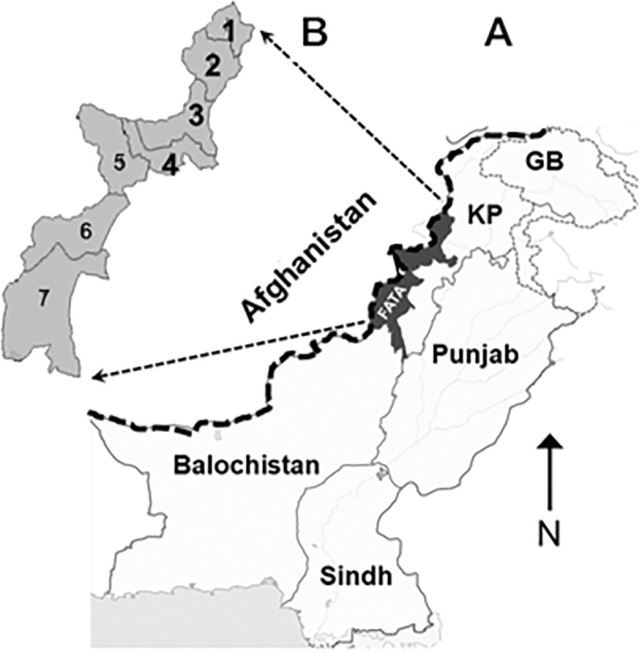
Map of Pakistan showing major provincial divisions **(A)** with a zoom-in map of FATA **(B)** depicting 7 districts from north to south: 1. Bajaur; 2. Mohmand; 3. Khyber; 4. Orakzai; 5. Kurram; 6. North Waziristan; and 7. South Waziristan. Data were collected from 4 northern districts (1–4) (modified from the source: https://worldmapblank.com/blank-map-of-pakistan/). FATA, Federally Administered Tribal Areas.

The Federally Administered Tribal Areas (FATA) is a tribal assemblage in north-west Pakistan, comprising 7 agencies (districts) stretching in a series at the Pakistan–Afghanistan mountainous border (**Figure 1**). It has an estimated population of 5 million individuals. Due to geopolitical unrest and war situations, FATA has remained highly impoverished and many of the socio-economic indicators are well below the national average; i.e., the rural population is 97% (compared to a national estimate of 64%), the poverty rate is 74% (compared to a national rate of 39%), the literacy rate is 33% (compared to a national rate of 60%), and persons with disability are 3.7% (compared to a national rate of 1.6%) [11, 12, 15].

Owing to the unique socio-cultural tribal norms in the area, the FATA region is a difficult place to approach for population-based studies. Traveling and accessibility are difficult in this mountainous territory without a local resource person and proper permission from the tribal heads who are authorized by the political administration to resolve disputes among tribesmen [12, 15]. War situation and mass migration (of IDPs) have an adverse impact on the health of the inhabitants. Post-traumatic stress disorders are common, particularly among children and women [10, 11, 12, 13]. The local people are not welcoming to the enumerators from outside, and hence, data collection and maneuvering in the area have been a profound challenge. Further, information gathering on the morbidity status of females is even more difficult due to the specific socio-cultural norms of the area [9, 14, 15]. Internal displacements and migrations have significant effects on the public health and well-being of IDPs. Several risk factors like overcrowding, malnutrition, lack of clean water, poor sanitation, and the outbreak of infections synergistically augment the occurrence of morbidities during displacements [16, 17]. During the flight and early settlement, women may be forced to give birth alongside roads, in forests, or in temporary shelters, resulting in complications of pregnancy and childbirth [16, 17, 18].

### Study participants recruitment

This cross-sectional clinico-epidemiological study was conducted in the upper FATA region (mainly Bajaur, Mohmand, Khyber, and Orakzai districts) of Pakistan between January 2017 and March 2019 (**Figure 1**) [15]. This study was approved by the Ethical Review Committee of Quaid-i-Azam University (certificate of approval No. DAS13/-). Approval was also taken from the tribal heads and local health authorities. All the data were acquired and documented in the presence of the family head or guardian after participants had provided informed written or formally documented verbal consent when illiterate. The cross-sectional reporting guideline of the Strengthening the Reporting of Observational Studies in Epidemiology (STROBE) statement was used [19].

Participating individuals or families (here referred to as participants or cases) with congenital and hereditary anomalies (here referred to as anomalies) were recruited through a combination of convenience sampling and cluster random sampling from hospitals, community centers (rural gathering places; *hujra*), and schools (or *madarsa*), and were brought to the nearest medical center for examination. There were at least 30 sampling sites covering the major towns or villages of 4 districts depending upon the availability of the local resource persons and the permission received from tribal heads. Each participant was physically examined by a local physician and the data on phenotypic, pedigree, and bio-demographic variables were collected on a structured proforma in face-to-face interviews. The fieldwork and data collection were performed by the first 2 authors who have formal training in medical genetics.

### Inclusion and exclusion criteria

To avoid any bias, the participants were recruited irrespective of their gender, ethnicity, origin, and type of anomalies. Only those participants were included that had a permanent residence in the study area. The anomalies with a confirmed congenital nature, hereditary nature, or both were included in the analyses. Participating individuals or families providing incomplete information were not included. Anomalies with accidental and traumatic natures were not included. The individuals with psychiatric or behavioral disorders were skipped. Muscle wasting with a likely poliomyelitis presentation was also skipped. Data were not collected from any special education school or disability center.

### Classification of anomalies, definitions and statistical methods

The initial assessment was provided by the specialist resident doctors and only the individuals with the confirmed diagnosis were recruited. Syndromic cases were identified with respect to the more severe symptoms in the following order: neurological disorders, neuromuscular defects, musculoskeletal defects, eye or visual impairments, sensorineural or ear anomalies, and limb defects [5, 14]. A pedigree comprising 3 or more generations was drawn for each case, but only the index participant from each family was considered in data analyses.

The definitions of anomalies were based on a standard coding system of the International Classification of Diseases (ICD-10; Ver.16), OMIM, and ORPHANET databases [20, 21, 22]. The patients with intellectual disability (ID) types were assessed according to the criteria of the American Psychiatric Association [23]. Limb defects were further characterized into minor entities [24, 25, 26]. Descriptive summaries were generated. For each anomaly, the proportions and 95% confidence intervals (95% CI) were estimated from the total number of anomalies. The analyses were carried out through GraphPad Prism (5.00) and Stata11.

## Results

A total of 361 independent participating individuals or families having certain types of anomalies were recruited. The index males were 74% (n = 267). The majority of the participants belonged to rural areas (n = 304; 84%), lived in extended or joint families (n = 288; 80%), and spoke Pashto (100%) (**Table 1**).

**Table 1.** Demographic attributes of recruited individuals

**Variable**	**Male**		**Female**		**Total**	

	**N**	**%**	**N**	**%**	**N**	**%**
Age range (years)*						
Up to 9	99	37.1	57	60.6	156	43.2
>9–19	110	41.2	29	30.9	139	38.5
>19	58	21.7	8	8.5	66	18.3
Total	267	100.0	94	100.0	361	100.0
Origin*						
Rural	226	84.6	78	83.0	304	84.2
Urban	41	15.4	16	17.0	57	15.8
Caste system						
Masozai	120	44.9	50	53.2	170	47.1
Tarkalani	49	18.4	18	19.1	67	18.6
Afridi	26	9.7	2	2.1	28	7.8
Uthman Khel	16	6.0	4	4.3	20	5.5
Others	56	21.0	20	21.3	76	21.1
Literacy level (age >5 years)						
Illiterate	127	55.9	46	64.8	173	58.1
Literate	100	44.1	25	35.2	125	41.9
Family type						
Nuclear	215	80.5	73	77.7	288	79.8
Extended or joint	52	19.5	21	22.3	73	20.2

* Differences in distribution were statistically highly significant.

The anomalies were grouped into 8 major groups (**Table 2**). With respect to their familial nature, the majority of the anomalies (76%) had sporadic presentations compared to 24% of familial cases. However, among the major groups, ectodermal anomalies and blood disorders were more often familial (*P* = 0.0001). Further, there was a high preponderance of anomalies with isolated presentations (76%), while 24% of cases were syndromic. However, neurological disorders were an exception where 51% of the cases had syndromic presentations (*P* = 0.0001).

**Table 2.** Distribution of major categories of anomalies with respect to familial or sporadic nature, isolated or syndromic presentations, and parental consanguinity

**Anomaly type**	**Total**		Familial or sporadic nature*		Isolated or syndromic*		Parental marriage type*	

	**N**	**%**	**Sporadic**	**Familial**	**Isolated**	**Syndromic**	**Consanguineous**	**Nonconsanguineous**
Neurological disorders	100	27.7	83	17	49	51	26	74
Sensorineural defects	70	19.4	48	22	63	7	33	37
Limb defects	60	16.6	51	9	52	8	14	46
Eye or visual impairments	55	15.2	46	9	48	7	18	37
Musculoskeletal defects	37	10.2	29	8	29	8	16	21
Ectodermal anomalies	13	3.6	4	9	8	5	7	6
Blood disorders	12	3.3	2	10	12	0	6	6
Others	14	3.9	13	1	14	0	3	11
**Total N (%)**	**361 (100)**	**100**	**276 (76)**	**85 (24)**	**275 (76)**	**86 (24)**	**123 (34)**	**238 (66)**

* Differences in distribution were statistically highly significant.

Parental consanguinity was observed in 34% of the cases. Among the major categories, consanguinity was prominent in ectodermal anomalies, blood disorders, and sensorineural defects (54%, 50%, and 47%, respectively) (**Table 2**). It is pertinent to mention that among the familial cases, the majority of the pedigrees were suggestive of autosomal recessive inheritance patterns (data not shown). The representation of index males was generally higher in the major categories. Further, in all families, the total number of affected participants was estimated to be 613 (males 403; females 210) (**Table 3**).

**Table 3.** Distribution of major categories of anomalies with respect to gender of index cases and total affected individuals in all families

**Anomaly type**	**Total**		**Index individual**		**Total affected in all families**		

	**N**	**%**	**Male**	**Female**	**Males**	**Females**	**Total**
Neurological disorders	100	27.7	79	21	42	25	67
Sensorineural defects	70	19.4	48	22	51	30	81
Limb defects	60	16.6	41	19	58	22	80
Eye or visual impairments	55	15.2	46	9	54	25	79
Musculoskeletal defects	37	10.2	26	11	46	22	68
Ectodermal anomalies	13	3.6	6	7	89	51	140
Blood disorders	12	3.3	11	1	53	30	83
Others	14	3.9	10	4	10	5	15
**Total N (%)**	**361 (100)**	**100**	**267 (74)**	**94 (23)**	**403 (66)**	**210 (34)**	**613 (100)**

* Differences in distribution were statistically highly significant.

Collectively there were at least 72 minor entities of anomalies (**Table 4**). Among the major categories, neurological disorders had the highest representation (n = 100; proportion: 0.277; 95% CI: 0.231–0.323), followed by sensorineural defects (n = 70; prop.: 0.194; 95% CI: 0.153–0.235), limb defects (n = 60; prop.: 0.166; 95% CI: 0.128–0.205), eye or visual impairments (n = 55; prop.: 0.152; 95% CI: 0.115–0.189), musculoskeletal defects (n = 37; prop.: 0.102; 95% CI: 0.071–0.134), ectodermal anomalies (n = 13; prop.: 0.036; 95% CI: 0.017–0.055), blood disorders (n = 12; prop.: 0.033; 95% CI: 0.015–0.052), and others (n = 14; prop.: 0.039; 95% CI: 0.019–0.059). A detailed distribution of minor entities and their relative proportions is presented in **Table 4**.

**Table 4.** Major and minor categories of anomalies, proportions, and classification

**Anomalies (major or minor)**	**N**	**Proportion**	**95% CI**	**ICD-10**	**OMIM**
**Neurological disorders**	**100**	**0.277**	**0.231–0.323**		
ID – all	58	0.161	0.123–0.199		
ID – mild	27	0.075	0.048–0.102	F70	249500
ID – moderate	18	0.050	0.027–0.072	F71	
ID – severe or profound	13	0.036	0.017–0.055	F72, F73	611091
Cerebral palsy	16	0.044	0.023–0.066	G80.9	605388
Down syndrome	11	0.030	0.013–0.048	Q90.9	190685
Epilepsy	6	0.017	0.003–0.030	G40	607208
Neuropathy	3	0.008	−0.001 to 0.018	G60	605253
Alzheimer disease	1	0.003	−0.003 to 0.008	F00.1	104300
Microcephaly	1	0.003	−0.003 to 0.008	Q02	251200
Multiple sclerosis	1	0.003	−0.003 to 0.008	G35	126200
Spastic paraplegia	1	0.003	−0.003 to 0.008	G82.1	182600
Spina bifida	1	0.003	−0.003 to 0.008	Q05	182940
Tremor	1	0.003	−0.003 to 0.008	G25.0	190300
**Sensorineural defects**	**70**	**0.194**	**0.153–0.235**		
Deaf–mute	48	0.133	0.098–0.168	H90	304500
Mute only	19	0.053	0.030–0.076	R47.0	
Stuttering	3	0.008	−0.001 to 0.018	F98.5	184450
**Limb defects**	**60**	**0.166**	**0.128–0.205**		
Talipes or clubfoot	13	0.036	0.017–0.055	Q66.9	119800
Limb amputations	12	0.033	0.015–0.052	Q73.8	217100
Polydactyly (poly.; all)	8	0.022	0.007–0.037		
Poly., preaxial type I	4	0.011	0.000–0.022	Q69.1	174400
Poly., postaxial type A	3	0.008	−0.001 to 0.018	Q69.0;Q69.2	174200
Poly., postaxial type B	1	0.003	−0.003 to 0.008	Q69.0;Q69.2	174200
Syndactyly (synd.; all)	8	0.022	0.007–0.037		
Synd., type 1c	3	0.008	−0.001 to 0.018	Q70.1	
Synd., type 1a	2	0.006	−0.002 to 0.013	Q70.3	609815
Synd., type II	2	0.006	−0.002 to 0.013	Q70.4	186000
Synd., type 1d	1	0.003	−0.003 to 0.008	Q70.2	
Contractures	5	0.014	0.002–0.026	M21.8	259450
Brachydactyly, 4th toe	2	0.006	−0.002 to 0.013	Q72.8	113475
Oligodactyly	2	0.006	−0.002 to 0.013	Q73.8	176240
Split-hand split-foot	2	0.006	−0.002 to 0.013	Q72.7	183600
Brachy-mesophalangy	1	0.003	−0.003 to 0.008		112800
Camptodactyly	1	0.003	−0.003 to 0.008	Q68.1	114200
Clinodactyly	1	0.003	−0.003 to 0.008	Q74.0	
Constriction ring	1	0.003	−0.003 to 0.008	Q79.8	217100
Leg length discrepancy	1	0.003	−0.003 to 0.008	Q72.9	
Overriding toe	1	0.003	−0.003 to 0.008		
Radial hemimelia	1	0.003	−0.003 to 0.008	Q71.8	114500
Ulnar hemimelia	1	0.003	−0.003 to 0.008	Q71.8	
**Eye or visual impairments**	**55**	**0.152**	**0.115–0.189**		
Squint eye (esotropia)	17	0.047	0.025–0.069	H50.0	185100
Squint eye (exotropia)	7	0.019	0.005–0.034	H50.1	
Blindness	14	0.039	0.019–0.059	H54.0	
High myopia	13	0.036	0.017–0.055	H52.1	160700
Anophthalmia	2	0.006	−0.002 to 0.013	Q11.1	
Color blindness	1	0.003	−0.003 to 0.008	H53.5	303800
Congenital nystagmus	1	0.003	−0.003 to 0.008	H55	617297
**Musculoskeletal defects**	**37**	**0.102**	**0.071–0.134**		
Dwarfisms	8	0.022	0.007–0.037	Q77.4	100800
Muscular atrophy	7	0.019	0.005–0.034	G12.1	253300
Muscular dystrophy	7	0.019	0.005–0.034	G71.0	310200
Kyphoscoliosis	4	0.011	0.000–0.022	M41.9	610170
Congenital hip dislocation	3	0.008	−0.001 to 0.018	Q65	142700
Kyphosis	2	0.006	−0.002 to 0.013	Q76.4	
Mucopolysaccharidosis	1	0.003	−0.003 to 0.008	E76.3	607014
Pectus carinatum	1	0.003	−0.003 to 0.008	Q67.7	245600
Pectus excavatum	1	0.003	−0.003 to 0.008	Q67.6	600399
Rickets	1	0.003	−0.003 to 0.008	E83.3	277440
Spinal muscular atrophy	1	0.003	−0.003 to 0.008	G12.1	253300
Torticollis	1	0.003	−0.003 to 0.008	M43.6	189600
**Ectodermal anomalies**	**13**	**0.036**	**0.017–0.055**		
Ectodermal dysplasia	3	0.008	−0.001 to 0.018	Q82.4	224900
Anonychia	2	0.006	−0.002 to 0.013	Q84.3	206800
Early tooth decay	2	0.006	−0.002 to 0.013	K02	
Ichthyosis	2	0.006	−0.002 to 0.013	Q80.1	602400
Albinism	1	0.003	−0.003 to 0.008	E70.3	300500
Dentinogenesis imperfecta	1	0.003	−0.003 to 0.008	K00.5	125490
Eczema	1	0.003	−0.003 to 0.008	L20	603165
Epidermolysis	1	0.003	−0.003 to 0.008	Q81.2	226600
**Blood disorders**	**12**	**0.033**	**0.015–0.052**		
Thalassemia (major = 8; intermedia = 1)	9	0.025	0.009–0.041	D56.1	613985
Hemophilia	3	0.008	−0.001 to 0.018	D66	306700
**Others**	**14**	**0.039**	**0.019–0.059**		
Cleft palate	4	0.011	0.000–0.022	Q35	119540
Heart septal defect	3	0.008	−0.001 to 0.018	Q24.9	600001
Lymphedema	2	0.006	−0.002 to 0.013	Q82.0	153100
Urogenital defect	3	0.008	−0.001 to 0.018	Q62	617641
Cleft lip	1	0.003	−0.003 to 0.008	Q36	119530
Enuresis	1	0.003	−0.003 to 0.008	R32	600631
**Total**	**361**	**1.000**	**1.000–1.000**		

CIs, confidence intervals; ICD-10, International Classification of Disease; ID, Intellectual disability; OMIM, Online Mendelian Inheritance in Man.

### Burden of neurological disorders

Among the neurological disorders (n = 100), ID types had the highest representation (58%), followed by cerebral palsy (16%), and Down syndrome (11%) (**Table 4**). The cases with ID were further resolved into mild, moderate, and severe or profound types and were observed to be n = 27, n = 18, and n = 13, respectively.

### Burden of sensorineural defects

In sensorineural defects (n = 70), most of the cases showed deaf–mute phenotype (69%), followed by mute only (27%) and stuttering (4%).

### Burden of limb defects

Among the limb defects (n = 60), there were at least 20 distinct phenotypic entities. Talipes and limb amputations had the highest representation (22% and 20%, respectively). Most of the cases of amputations were transverse and unilateral and were the cause of severe disability (data not shown).

### Other anomalies

Among the eye or visual impairments (n = 55), there were at least 7 entities, and squint eye types were the most prominent anomaly (44%), represented as esotropia or exotropia. Musculoskeletal defects were represented by at least 12 distinct entities, and of those, dwarfism was the most common type. Among the ectodermal anomalies, there was a representation of 8 minor types. A detailed distribution of major and minor anomalies is presented in **Table 4**.

### Associations among the syndromic cases

Among the syndromic cases, neurological disorders were most prominent (n = 48), followed by limb defects and eye or visual impairments. The most common associated anomaly was deaf– mute (n = 35), followed by eye or visual impairments (n = 16). The combinations of associated anomalies are depicted in **Table 5**.

**Table 5.** Syndromic cases with a combination of associated anomalies

**Major anomaly**	**Associated anomaly**											

	**Deaf–mute**	**Eye or visual impairments**	**Cerebral palsy**	**Epilepsy**	**Growth retardation**	**Polydactyly**	**Club foot**	**Oligodactyly**	**Contracture**	**Syndactyly**	**Others**	**Total**
Neurological disorders	30	8	7	1							2	48
Sensorineural defects					1					1	5	7
Limb defects						1	2	2	2		2	9
Eye or visual impairments	2	5			1						1	9
Musculoskeletal defects	2	2					1				3	8
Ectodermal defects		1									2	3
Others	1											1
Total	35	16	7	1	2	1	3	2	2	1	15	85

## Discussion

This study presents the broad spectrum of anomalies in the north-west territories at the Pakistan–Afghanistan border. The majority of the anomalies were of severe nature and a cause of disability in the individuals. In this cohort, the category of neurological disorders, including ID, was the most prevalent. This observation is similar to the one drawn in Hussain et al.'s study [3], wherein it was discovered that in a cohort of 226 neonates monitored at a tertiary care hospital in Kharian, Pakistan, the anomalies related to the central nervous system were most common (20%). In a similar study carried out by Perveen and Tyyab [4] in Karachi, Pakistan, neural tube defects were found to be the commonest (66%). Among the participants with ID in our study, the cases with mild ID types were more frequent, followed by moderate and severe ID types. These findings are similar to Koirala et al.'s study [27], in which also a high prevalence of mild ID was reported, followed by moderate, severe, and profound types. Further, the proportion of sensorineural defects was 19% and it constituted the second most prevalent category. In many of the reported epidemiological studies, neurological disorders and sensorineural defects show high representations, which could be because both these organ systems require an extended period during embryonic development and any perturbance during this period may cause morbidity [28].

Limb defects comprised 17% of our cohort. Curiously, among the limb defects, talipes and limb amputations had the highest representations (22% and 20%, respectively). In the experience of Bhatti et al. [29], who conducted an epidemiological study in the population of the Sialkot district of Pakistan, clubfoot and polydactyly types were the most prevalent types of limb defects. However, in a study carried out in Chitral, Pakistan, talipes and limb amputations comprised only 3% each of the total limb or musculoskeletal anomalies, whereas polydactyly was the most common [30]. In another study conducted in the Sindh region of Pakistan, talipes and limb amputations were 2% and 8%, respectively, of the total limb anomalies [31]. A very high preponderance of such defects in our cohort could be a strong indication of nongenetic etiological factors and complications during pregnancy [32].

In the present study, most of the anomalies had sporadic occurrence (76% compared to 24% familial cases). A marked variation, however, was evident in the sporadic or familial presentation among the major categories. Here, the sporadic occurrence was common in neurological disorders, sensorineural defects, limb defects, eye or visual impairments, and musculoskeletal defects, whereas ectodermal anomalies and blood disorders were familial most often. Concordantly, in the experience of Zahra et al. [14] and Bhatti et al. [29], the sporadic occurrence was common in limb defects, neurological disorders, musculoskeletal defects, and sensorineural defects assembled from Kurram and Sialkot, Pakistan.

In the present sample, the parental consanguinity was 34%, which is very low compared to the baseline consanguinity rate of 57%–62% reported for other Pakistani populations [6, 7, 8, 33]. The differences in the distribution of consanguineous and nonconsanguineous unions among the major categories of anomalies were statistically significant (*P* < 0.0001). The rate of consanguinity was higher in the ectodermal anomalies, blood disorders, and sensorineural defects (54%, 50%, and 47%, respectively). The potentially low rate of parental consanguinity is similar to the observations recorded in Bhatti et al.'s study [29], in which a parental consanguinity rate of 17% was ascertained in a cohort of anomalies assembled from the population of Sialkot, Pakistan. Concordant to our study, Bhatti et al. [29] also observed that the differences in the distribution of consanguineous and nonconsanguineous unions among the major categories of anomalies were statistically significant. Further, the rate of consanguinity was higher in subjects with neuromuscular anomalies and neurological disorders (30% and 21%, respectively), and significantly higher in familial cases compared to sporadic ones. In our data, the low rate of consanguinity may also indicate the potentially high role of nongenetic factors in the etiology of these anomalies.

The current study has several limitations. First, the true prevalence rate of each anomaly has not been established. Due to various reasons, it was not possible to launch a true cluster or stratified sampling encompassing the whole area. We, therefore, primarily relied on a combination of convenience sampling and cluster random sampling, which was facilitated by the local resource persons. Second, the recruited anomalies were generally typable through physical examination and non-invasive medical investigation. The anomalies requiring invasive diagnostic characterization and detailed biochemical tests may be underrepresented in this sample. Further, prenatal and postnatal mortality, maternal morbidities and pregnancy conditions, and potential risk and etiological factors remain to be elucidated. The findings of this study may not be generalizable to the rest of the Pakistani population.

This study has several strengths. It is the first report of anomalies prevalent in the war-affected territory of the upper FATA region. In Pakistan, in general, there is no systematic monitoring and compilation of anomalies and there is currently no national database of anomalies. The surveillance of anomalies is difficult because of inadequate funding, poor infrastructure, and the lack of trained staff and enumerators. It is almost impossible to evaluate the potential risk factors and to implement effective prevention and management services without detailed epidemiological data. In this context, the present study is a pilot effort to record and document anomalies prevalent in the population. These data would be very valuable for assigning priorities, resource allocation, and establishing management systems for these disorders.

## Conclusion

The high incidence of neurological, sensorineural, and limb disorders, the preponderance of cases with sporadic nature, and the relatively low level of parental consanguinity may indicate a likely high contribution of environmental factors in the etiology of these anomalies. Further, the high occurrence of talipes and limb amputations among the limb defects may also clue toward the role of nongenetic factors. Further, it is quite likely that continued warfare might have added environmental pollutants, residues from explosives, and heavy metals to the human food chain [34]. Further studies are warranted to understand the role of nongenetic factors in the etiology of anomalies and there is a dire need for the management of the anomalies.
